# Perceptions of the causes of eating disorders: a comparison of individuals with and without eating disorders

**DOI:** 10.1186/2050-2974-1-S1-P12

**Published:** 2013-11-14

**Authors:** Elizabeth Blodgett Salafia, Emily Haugen, Shauna Erickson

**Affiliations:** 1 North Dakota Room: State University, USA

## 

People's perceptions of the causes of eating disorders are important to identify in order to correct misconceptions. A few studies interviewed individuals with eating disorders (Button & Warren, 2001; Dignon et al., 2006; Lacey et al., 1986), but were limited by small samples and inconsistent findings. Although some studies surveyed the general public (Mond et al., 2004; Smith et al., 1986), only one compared individuals with and without eating disorders by asking them to select causes from pre-identified factors (Holliday et al., 2005). In this study, we sought to obtain greater depth in understanding people's perceptions.

Our sample included 57 individuals with eating disorders and 220 without. Participants responded to open-ended questions about the causes of eating disorders. Eight causal categories were determined.

Participants with eating disorders identified causes at different rates. Psychological/emotional and social problems were most frequently endorsed, with genetics/biology and media/culture ideals least endorsed. No significant relationships existed between types of eating disorder and causes specified. Additionally, participants without eating disorders identified causes at different rates. Psychological/emotional problems and media/culture ideals were most frequently endorsed, with traumatic life events and sports/health least endorsed. These samples differed in their perceptions, suggesting that misconceptions exist in the general public.

